# A hip that breaks the scale

**DOI:** 10.1016/j.ero.2026.03.019

**Published:** 2026-04-18

**Authors:** Nils Schulz, Tim Wilhelmi, Pascal van Wijnen, Ulf Müller-Ladner, Philipp Klemm

**Affiliations:** Department of Rheumatology, Clinical Immunology, Osteology and Physical Medicine, Campus Kerckhoff, Justus-Liebig-University Gießen, Bad Nauheim, Germany

## PART 1

A 68-year-old woman presented with progressive unilateral left thigh/hip pain. The pelvic radiograph shows pronounced remodelling of the left proximal femur with coarse trabeculation, sclerosis, and cortical thickening, while the contralateral femur appears comparatively unremarkable (Figure B). Dual-energy X-ray absorptiometry (DXA) performed for bone health assessment demonstrates extreme site discordance: areal bone mineral density (BMD) is markedly increased at the left total hip (BMD 1.831 g/cm²; T-score +6.9), whereas the right hip is near normal (BMD 0.950 g/cm²; T-score −0.4) and the lumbar spine is osteopenic (L1–L4 T-score −1.2) (Figure A). Laboratory testing indicates increased level of bone turnover with alkaline phosphatase 127 U/L (reference 35-104) and elevated level of bone-specific alkaline phosphatase 34.5 µg/L (reference <22.4), whereas calcium, 25(OH) vitamin D, and C-reactive protein levels are normal. Bone scintigraphy demonstrates focal increased tracer uptake confined to the left proximal femur.

**Figure:**
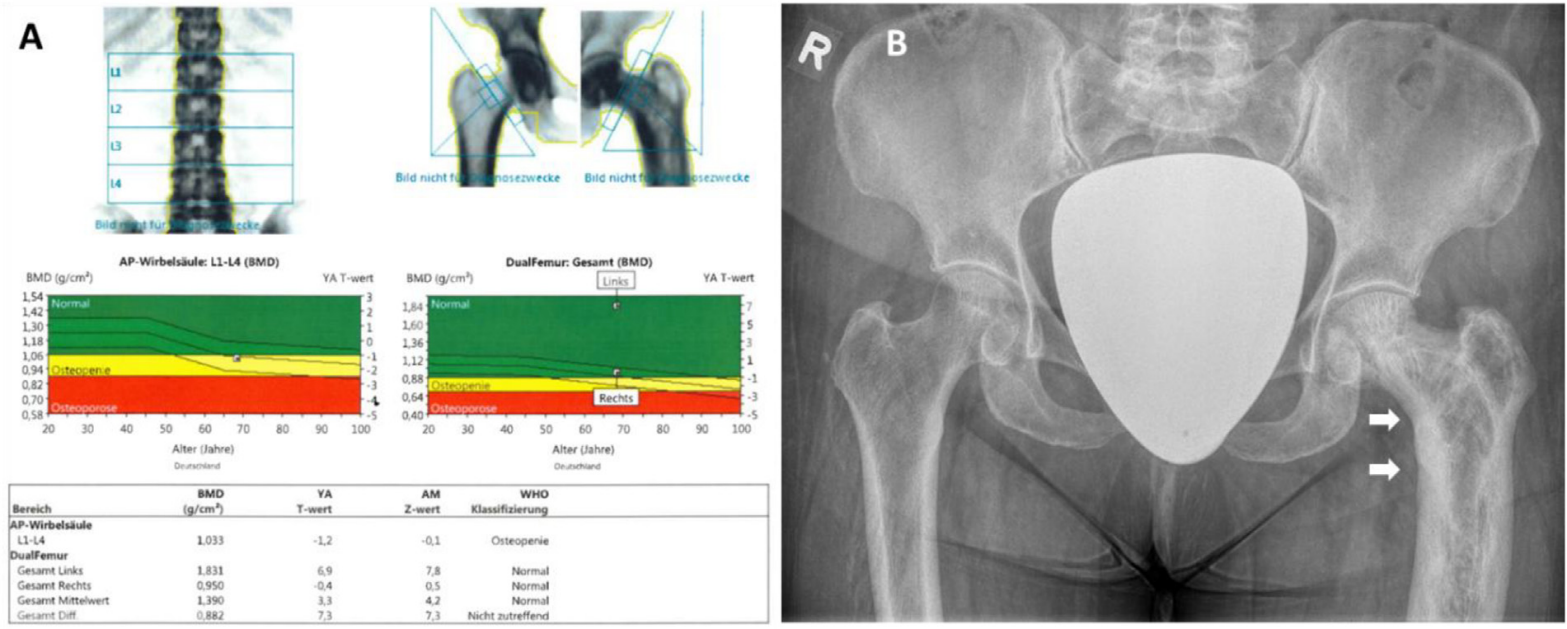
A, Dual-energy X-ray absorptiometry demonstrating extreme site discordance with markedly increased areal BMD at the left total hip compared with the right hip, contrasted by osteopenic lumbar spine values. B, Pelvic radiograph highlighting focal osseous remodelling of the left proximal femur (arrows). BMD, bone mineral density.

Which diagnosis best explains this paradox of ‘supernormal’ hip BMD at the painful site, and why can a very high hip T-score be clinically misleading?

## PART 2

The pattern is most consistent with Paget disease of bone involving the left proximal femur, while fibrous dysplasia is less likely given the patient’s age and the characteristic imaging features of coarse trabeculation, sclerosis and cortical thickening [1]. Pagetoid remodelling can markedly elevate areal BMD on DXA, producing a misleadingly high T-score that reflects local pathology rather than ‘exceptional’ bone strength [2]. Importantly, despite high areal BMD, pagetic bone may be mechanically fragile, predisposing to local pathological fractures in affected weight-bearing sites [1]. Therefore, the affected hip should be excluded from osteoporosis classification and risk interpretation. Unaffected sites (contralateral hip and/or lumbar spine) are more appropriate [2]. After zoledronate, pain improved and alkaline phosphatase level declined to 53 U/L at 1 year.

## CRediT authorship contribution statement

**Nils Schulz:** Writing – original draft, Visualization, Conceptualization. **Tim Wilhelmi:** Writing – review & editing. **Pascal van Wijnen:** Writing – review & editing. **Ulf Müller-Ladner:** Writing – review & editing. **Philipp Klemm:** Writing – review & editing, Conceptualization.

## Competing interests

All authors declare they have no competing interests.

## Appendix Supplementary materials

Image, application 1
