# Supplementary material for: A hip that breaks the scale
Source: EULAR Rheumatol Open. 2026 Apr 18;2(2):100171. doi: 10.1016/j.ero.2026.03.019 (PMC13425170; doi:10.1016/j.ero.2026.03.019)
Supplement: Supplementary file 1 [file mmc1.pdf]

Patient's Consent to Publication

Title of product: A hip that breaks the scale

Author: Nils Schulz, Tim Wilhelmi, Pascal van Wijnen, Ulf Mueller-Ladner, Philipp Klemm

Fig. no. and caption: Figure 1

This is to state that I give my full permission for the publication, reproduction, broadcast and other use of photographs, recordings and other audio-visual material of myself (including of my face) and textual material (case histories) in all editions of the above-named product and in any other publication (including books, journals, CD-ROMs, online and internet), as well as in any advertising or promotional material for such product or publications.

I declare, in consequence of granting this permission, that I have no claim on ground of breach of confidence or any other ground in any legal system against — Nils Schulz — and its agents, publishers, successors and assigns in respect of such use of the photograph(s) and textual material (case histories).

I hereby agree to release and discharge Nils Schulz, and any editors or other contributors and their agents, publishers, successors and assigns from any and all claims, demands or causes of action that I may now have or may hereafter have for libel, defamation, invasion of privacy, copyright or moral rights or violation of any other rights arising out of or relating to any use of my image or case history.

Name: Dagmar Weber-Henke

Address: Tornowstraße 65, 60486 Frankfurt am Main, Germany

Signed: D. Weber-Henke

Date: 19.12.2025
